# Youth living with HIV/AIDS in secondary schools: perspectives of peer educators and patron teachers in Western Uganda on stressors and supports

**DOI:** 10.1080/17290376.2019.1626760

**Published:** 2019-06-09

**Authors:** Emmanuel Kimera, Sofie Vindevogel, John Rubaihayo, Didier Reynaert, Jessica De Maeyer, Anne-Mie Engelen, Johan Bilsen

**Affiliations:** a School of Health Sciences, Department of Public Health, Mountain of the Moon University, Fort Portal, Uganda; b Faculty of Education, Health and Social Work, University College Gent, Gent, Belgium; c Department of Public Health, Vrije Universiteit Brussels, Brussels, Belgium; d Faculty of Education, Health and Social Work, Department of Orthopedagogy, University College Gent, Gent, Belgium; e School of Health Sciences, Mountains of the Moon University, Fort Portal, Uganda; f Faculty of Education, Health and Social Work, Department Social Work, University College Gent, Gent, Belgium; g Faculty of Education, Health and Social Work, Centre of expertise on Quality of Life, University College Gent, Gent, Belgium; h Faculty of Education, Health and Social Work, Department of Occupational therapy, University College Gent, Gent, Belgium; i Department of Public Health, Mental Health and Wellbeing research group, Vrije Universteit Brussels, Brussels, Belgium

**Keywords:** HIV/AIDS, stressor, support, school, stigma, youth

## Abstract

As Youth Living With HIV/AIDS (YLWHA) continue to survive and live with HIV chronically due to effective Antiretroviral Therapy (ART), it is paramount to work toward maximising their psychosocial wellbeing. The school where these YLWHA are expected to spend most of their time is an excellent environment to investigate this. In this study, we explore perspectives of Peer Educators (PEs) in secondary schools of one district in Western Uganda on how YLWHA are perceived in school, on their daily stressors and their way of coping with their HIV-positive serostatus given the support of the schools. We conducted eight focus groups with a total of 59 students who were members of Peer Educators Clubs (PECs) as well as 8 in-depth interviews with patron teachers of PECs in eight secondary schools of Kabarole district, selected through a stratified random sampling method. Focus groups and interviews were tape-recorded, transcribed and analysed thematically both inductively and deductively. Stressors and support in schools, as identified by the PEs were categorised into three interrelated thematic domains; psychological wellbeing of YLWHA, disclosure of HIV status by YLWHA, and health and treatment adherence. Stigma was found to be a key stressor and an intermediary in all the three thematic domains Stressors affecting psychological wellbeing were fear of death and uncertainty of the future compounded by financial and academic challenges. Stressors affecting disclosure centred around lack of privacy, confidentiality and fear of loss of friends. Stressors affecting treatment adherence included lack of privacy while taking drugs, unintended disclosure while obtaining drugs or seeking permission to attend clinic appointments and fear of drug adverse effects due to poor nutrition. A supportive school environment involved the availability of a school nurse, counselling services and PECs. We conclude that the school environment brings more stressors than supports for YLWHA. The daily stressors related to HIV stigma, uncertainty, disclosure, privacy and confidentiality render schooling a hassle for YLWHA. Interventions that promote resilient school communities are necessary to foster disclosure in a non-discriminatory and stigma-free environment. This calls for concerted efforts from all school stakeholders.

## Introduction

Globally youth are at the epicentre of the Human Immunodeficiency Virus and Acquired Immune Deficiency Syndrome (HIV/AIDS) pandemic with almost half of all new infections in people aged 15–24 years (Monascha & Mahyb, 2006; Wilson, Wright, Safrit, & Rudy, 2010). Uganda counts approximately 170,000 Youth Living With HIV/AIDS (YLWHA) (UNAIDS, 2014) and this group continues to feature among those vulnerable to new HIV infections (Schuyler et al., 2017). Kabarole district, the site for this study has one of the highest HIV prevalence in the general population at 11.3%, which is 5.8% higher than the average of the Western Uganda where the district is located and 5% higher than the national HIV prevalence of Uganda (Kabarole DHO, 2017; WHO, 2017). Many of these are youth vertically or behaviourally infected (Vu et al., 2017).

Getting infected or being disclosed to, the seropositive status can be an emotionally stressful or even a traumatic event for youth (Martin & Kagee, 2011; Olley, Zeier, Seedat, & Stein, 2005; Sherr et al., 2011; Vreeman et al., 2010), because HIV/AIDS is historically associated with death and it is highly stigmatised (Logie & Gadalla, 2009; Sawyer, Drew, Yeo, & Britto, 2007; Vanable, Carey, Blair, & Littlewood, 2006). In a Kenyan study, Gachanja (2015) described the post-disclosure experiences of HIV-positive school going children as shocking, disheartening and enraging. Therefore, being diagnosed with HIV/AIDS can induce a psychosocial burden in youth with an enormous impact on their daily life like participation in school, performance at work and later their socio-economic status in society. Further, post-diagnosis or post-disclosure health challenges, ongoing treatment, stigma and discrimination, as well as changes in future plans and expectations can evoke significant ongoing distress that continues to substantially challenge the wellbeing of YLWHA (Kyngäs, 2004). These daily stressors emerge from social and material conditions of everyday life in settings wherein the youth participate (Miller, Fernando, & Berger, 2009).

A major daily stressor featuring in previous studies with YLWHA is stigma (e.g. Block, 2009; Lee, Kochman, & Sikkema, 2002; Mutumba et al., 2015; Ramaiya et al., 2016; Tsai et al., 2013; Vanable et al., 2006). Stigma intersects strongly with feelings about the self, social behaviour and health outcomes (Quinn & Earnshaw, 2013). Living with HIV/AIDS might be complicated considerably when the environment fails to respond resiliently to disclosure, stigma and emerging special needs of YLWHA. Hence, when providing adequate support and access to resources, supportive environments may work towards maximising youths’ psychosocial wellbeing and protecting them against the full impact of HIV/AIDS on their lives (Amzel et al., 2013; Miller et al., 2009; Vreeman et al., 2010).

This study focuses on the school as a significant environment for YLWHA (Abubakar et al., 2016; Roeser, Eccles, & Sameroff, 2000) and examines the daily stressors and supports it produces for them. Since several international and local policies promote school inclusion by advocating for education for all (Ghergut, 2011) and since the universal education policies in many Sub-Sahara African (SSA) countries like Uganda removed the monetary barricade and stimulated increased school enrolment and retention (Chapman, Burton, & Werner, 2010; UNICEF, 2003), school communities have an enormous potential and obligation towards supporting their students in vulnerable situations, such as YLWHA. In a study conducted in Uganda, Birungi et al. (2008) found that many HIV-positive youth were schooling and desired educational achievements.

Additionally, the Presidential Initiative on AIDS Strategy for Communication to Youth (PIASCY) policy for Uganda (2002), and Education and Sports Sector National Policy Guidelines on HIV/AIDS (2006), led to the creation of Peer Education Clubs (PECs) in schools of Kabarole district in 2008. The PECs have several objectives that include providing in-school peers with Sexual and Reproductive Health (SRH) information and counselling that anticipates and meets their needs as well as encouraging peers through education and awareness to take health-promoting decisions regarding their SRH. These PECs are headed by a patron teacher in the school and have free membership that is open to all students. The members receive training from the district PEC team on the content and methodology to disseminate SRH information to their peers. Since the PECs are the closest in-school supportive structures for YLWHA, they can provide a valuable perspective on the daily stressors and supports YLWHA experience within a school environment. Moreover, because of their position and mandate in Ugandan schools, PECs can ably assess the nature of environment schools are or can be to YLWHA. Gaining knowledge on how these supportive networks of peers perceive the plight of YLWHA in the school context can therefore advance the understanding of how supportive school environments can be further developed, with important implications for policy and design of school-based interventions for YLWHA. The objective of this study was hence to investigate perceptions of PE students and patron teachers in Kabarole district on the daily stressors YLWHA face in school as well as the current and future supports that schools can provide to improve the wellbeing of YLWHA. Through perspectives of PE, we intended to answer the question: what hampers or facilitates living with HIV for youth in school communities of Uganda?

## Methods

### Study design and participants

This study is part of a larger research project with a goal of developing a sustainable intervention to improve the wellbeing of YLWHA in schools and larger communities of Kabarole district in Western Uganda. The current study was qualitative, using focus groups and interviews, and was conducted in eight secondary schools within this district from April to July 2017. We used stratified random sampling to select the schools based on an updated list of all secondary schools obtained from the Education Office of the district. All the schools were grouped into two strata; rural and urban according to where they were located. In these two strata, schools were further grouped as public or private depending on ownership. Finally, the public and private schools were further grouped into day schools and boarding schools. One school was randomly selected from the 8 formed strata using a rotary method. We involved a total of 67 participants, 59 of whom were PE students with the age range of 12–19 years and 8 were patron teachers of PEs. Of the 59 PE students, 33 were females.

### Data collection procedure

In each of the eight selected schools, the Headteachers were approached and the purpose of the study was explained to them. On consenting, the headteachers identified the patron teachers for the PECs who we then approached. After we had explained the purpose and procedure of the study, we sought verbal consent from the patron teachers who then convened an average of 8 PE students. We guided patron teachers to select PE of varied ages ranging from 12 years to 19 years and of different sexes. We also provided detailed information to these students and sought their willingness to participate in an audio recorded discussion. One in-depth interviews with the patron teacher of PEC and one focus group discussion (FGD) with student members of PEC were conducted in English, in each selected school, in a private quiet room identified by the patron teacher to ensure privacy and confidentiality. Additionally, we cautioned participants in FGDs not to share information discussed in the group with other students in school. We did not consider age stratification in all FGDs and sex stratification in FGDs conducted in mixed-sex schools to ensure homogeneity because the participants were already interacting as PEs and therefore could freely discuss. The first author (EK), a trained teacher with 10 years’ experience of working with youth in Ugandan secondary schools, conducted all FGDs and the interviews. Interviews and FGDs were conducted in English because it is the official language and medium of instruction in Ugandan schools. Interview and focus group Guides with semi-structured questions were used, focusing on; (1) perceived challenges that YLWHA experience in the school community; (2) perceived solutions to ameliorate the challenges now or in the future; and (3) inspiring practices in schools to deal with the phenomenon of YLWHA. By use of appropriate probes, depth of meaning and description was obtained. These questions were influenced by the systematic literature review we conducted on this topic (see Kimera et al., 2019). The focus groups with PE students and interviews with patron teachers of PECs were audio-recorded and lasted between 1 and 1½ h. Participants were invited to think about YLWHA they knew in order to answer questions from the third person’s perspective, thus findings are reported as such. The patron teachers were interviewed after the FGD to prevent their knowledge of interview guidelines biasing selection of students for the FGDs. We were oblivious about the HIV status of all the participants. We conducted the interviews and discussions during extra-curricular social or club time in the schools to minimise disruption of school activities. In addition to the audio recorder, a notebook was used to record non-verbal characteristics of the groups such as number of participants, sex and group interactions as well as new issues emerging in the discussions. Following the focus groups and interviews, audio recordings were transcribed verbatim. Names and identities of participants were anonymized. No information came up during the discussions that indicated any form of distress to participants that urged for follow-up. The study protocol was also reviewed and approved by the ethics committees of The AIDS Support Organization (TASO) and the National Council of Science and Technology in Uganda as well as the Ethical Committee of the University Hospital of Brussels, Belgium.

### Data analysis

Both inductive and deductive approaches to thematic analysis were involved (Braun & Clarke, 2006). Preliminary analyses started during data collection by note taking and reflections on the data collected to establish the point of saturation. We initially planned to conduct interviews in 6 schools but by the 6^th^ school, new themes were still emerging. Themes started to repeat by the 7th school which compelled us to involve one more school to confirm data saturation and thus the 8 schools involved.

In the final analysis, EK listened to the audio recordings and read transcripts several times to get immersed in the data. Inductive open coding of 4 transcripts (2 individual interviews and 2 FGDs) followed involving EK and SV working independently. The developed codes were then shared and through consensus, an initial coding scheme was developed. EK then used this scheme to code the remaining transcripts but remained open to new codes that arose in due course. Next, the codes were arranged into main themes and thematic domains that were derived deductively basing on the research question and the theoretical perspective of the study developed through a systematic literature review. Main themes were created in an iterative manner by grouping codes to create typologies while maintaining a trail of the source transcript and line numbers of the coded data in a matrix using an excel sheet. Finally, associations between themes in a thematic network were defined.

## Findings

**Figure 1.**
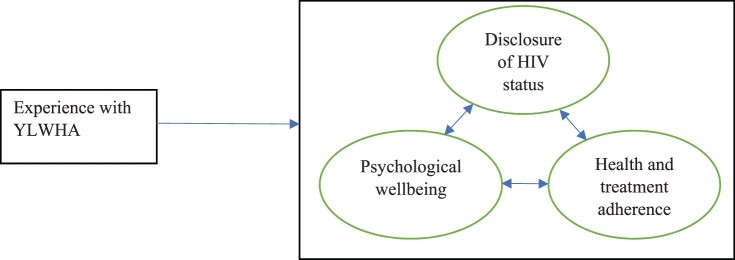
Domains in which YLWHA are stressed and supported in school.

Results from FGDs and individual interviews were integrated because the same themes were derived from both data sources and are thus reported together. Based on the analysis of experiences and perspectives of PEC members and patron teachers, we situate the daily stressors and supports for YLWHA in schools into three major interrelated domains: disclosure of HIV status, psychological wellbeing, health and treatment adherence as shown in Figure 1 below.

### Psychological wellbeing

#### Stressors

Participants perceived YLWHA in their school community as having to cope with psychological distress resulting from both the emotional burden of being seropositive and the stigmatising context they interacted with. In their discourses, it was noted that all types of stigma would be experienced by YLWHA. The fear of being known as living with HIV and of other peoples’ reactions following disclosure was told to lead to perceived stigma. Enacted stigma was mainly noted in discrimination and unfair treatment from others at school following disclosure and in loss of friendships. PEs mainly noted demoralisation and overthinking in YLWHA, as expressed in feeling sad, feeling that they are different from others, feeling out of place and thinking about death all the time. In the participants’ perception, this would lead to depression, self and community isolation, loneliness and loss of hope. Participants argued that YLWHA would see no value in schooling as a result of their evaluation of a discounted life due to HIV/AIDS. This would lead to poor academic performance as well as dropping out of school, which was often observed by the PEs. They also experienced that the discrimination, feeling of unworthiness and hopelessness impacted negatively on the behaviour of YLWHA. They would engage in unbecoming behaviours within school and outside school in a way of venting their bitterness.

That person, if she knows that she has the disease, she would start saying that for me now my life has ended here. She may even stop school and say let me go and stay somewhere else because she does not want to give the disease to other people. (Male, PE student)

They [students] are now associating with them [YLWHA] because they don’t know what they really are [that they have HIV/AIDS] but if they know, they may not even want to share anything with them. (Female, patron teacher)

The study pointed to the lack of adequate resources and support mechanisms in place within the school context to tackle the stigma and hence prevent psychological distress in YLWHA. For instance, PE students shared their observation that teachers who may get to know about a student with the seropositive status would shy away from talking to them about it, fearing to reinforce the stigma or adding a psychological burden when addressing it publicly. It was additionally noted that stigma would cause some teachers to desist from talking about HIV/AIDS in class fearing to offend those students who may already be infected. HIV prevention messages that were dispersed at school were also judged by participants as potentially stigmatising. The messages that teachers believe are necessary to elicit fear in youth so that they abstain from sex and avoid getting HIV were believed by participants to devastate those that already have HIV. Participants noted that messages like ‘if you have HIV/AIDS you are finished’ or ‘HIV/AIDS kills’ were common from teachers and also on signposts in the school compound. This is normally done in consistence with the PIASCY policy strategy that promotes abstinence-only for in-school young people.

Another challenge is when a teacher comes to class and starts talking about HIV, like you people protect yourself so that you don’t get HIV, if that person [YLWHA] is in class he gets embarrassed and feels like maybe the world is no more because even the teacher can say that if you have HIV just expect to die. So, if such a person is in class, he feels demoralized. (Female, PE student)

PEs further identified stressors emerging from other settings, affecting the psychological wellbeing and support needs of YLWHA in school. Parental loss is such a stressor that YLWHA would carry with them to school, especially those that were perinatally infected and are currently living in child-headed households or with extended family. According to participants, such YLWHA would lack parental care and they would also encounter financial stress to attend school. The misconceptions about HIV and YLWHA prevalent in society would also impacting strongly on how YLWHA are perceived and dealt with by other people, creating a huge source of psychological distress as well as affecting how they feel about themselves in a social setting like school. For instance, PEs noted that society believes that youth cannot get HIV. This would puzzle YLWHA as they wonder how they got HIV and why it had to be them. The other common misconception reported by PEs is that female youth get HIV through prostitution. This would lead to intersectional stigma in female YLWHA since prostitution is another stigmatised identity.

… in the community if they get to know that a young person has HIV/AIDS, they start talking about that person and the whole community gets to know. The community people always think that whoever has HIV is a Malaya [prostitute]. (Female, PE student)

some people in the community may feel sad that this young person is going to die soon but some may simply say let that girl die, she has been moving out with many boys even if you do not have any boyfriend. (Female, PE student)

Daily stress from academic work was a common theme from participants. The PEs noted that since YLWHA need more time to rest and frequently fall sick, they would be mentally overstretched by performance pressures at school. They also alluded to the tight time schedules in school that would not allow for enough time for YLWHA to rest.

#### Supports

Participants envisaged psychological support for YLWHA in the school, and motivated that YLWHA can find comfort, counselling and advice in carefully selected supportive confidants among their peers or school staff. Participants additionally see the school as having potential to equip all learners including those with HIV with knowledge and life skills through mentorship discussions, clubs such as peer education clubs, regular guidance and counselling from teachers as well as sensitisation from external HIV/AIDS support organisations that would be invited to school once in a while. For continued school attendance, participants suggested that schools would provide fee waivers for YLWHA.

The whole school should understand how to treat people with HIV. It starts with attitude and in our school that is handled during counselling sessions with the school counsellors, the mentorship classes, those activities we have. Such activities to change people’s attitude towards those people’s problems because we need to understand them so as to help them in the best way possible. (Male, PE student)

Despite being a stressor, participants argued that the activity packed school environment would provide the necessary distraction to YLWHA, thereby curtailing their overthinking and negative thoughts about the impact of HIV on their life.

### Disclosure of HIV status by YLWHA

#### Stressors

Following that participants viewed the school environment as one where stigma and discrimination are prevalent daily stressors for YLWHA, they argued that perceived and self-stigma that often comes with HIV/AIDS would hinder YLWHA from disclosing their status to peers, teachers and school authorities for fear of discrimination. PE’s portrayed the school environment and especially boarding schools as lacking privacy due to congestion, which often led to inadvertent disclosure. YLWHA were portrayed as continuously living in fear that their status would be involuntarily disclosed with consequences of enacted stigma and discrimination. For instance, within dormitories, fellow students would easily come across the drugs or medical records of YLWHA.

In the school, there are many youths with different behaviour. If they get to know that this one is positive, they start laughing at him of which it is bad. In the community outside school, there are more old people who understand the condition. (Female, PE student)

The school setting was by participants also characterised as lacking confidentiality. It was noted that if a YLWHA disclosed to a friend, it often happens that friends tell other friends and, in that way, the entire school would know. Stigma would then be enacted as loss of friendship, segregation and discrimination from group activities.

Romantic relationships were also viewed as stressful for YLWHA since they urge the disclosure dilemma and could easily lead to inadvertent disclosure and rejection by potential partners, even with self-disclosure. Misconceptions in the society which also influence schools’ approach to YLWHA also limit the disclosure. One such belief noted by PEs is that other people can contract HIV though social interactions like games with people living with HIV.

When they disclose, they start being isolated. Historically someone with AIDS even if it was a member of staff here, it is terrible. People would look at you and think they will contract the disease even when they play with you these games like football or netball. If is still feared. (Male, patron teacher)

In relation to this, the lack of confidentiality and professionalism by school staff, specifically the teachers and school nurses, also featured as a stressor. It was stated that on learning about the status of YLWHA, some teachers could disclose this information amidst other students as an insult when YLWHA misbehave. This would then cause YLWHA to be stigmatised and discriminated. Participants suggested that YLWHA would easily disclose to professional counsellors who sporadically come to school because they are trained to keep confidential information, unlike teachers. The patron teachers of PECs, however, denied this, stating that it was against their code of conduct to disclose confidential information about their students.

Apart from teachers, secondary schools also have a school nurse tasked with a responsibility of keeping drugs for all students on medication to ensure adherence. According to participants, YLWHA would also find this a distressing factor at school. Other students would easily suspect the status of YLWHA due to regular visits to the school nurse to take their medicine and unique type of medicine they would be taking in addition to frequent illnesses and visible body signs. Moreover, it was reported that nurses are often a potential source of unintended disclosure when they identify the medicine as HIV drugs and at the time of giving it to YLWHA in the presence of other students.

You find that, may be someone has not accepted the fact that they are living with HIV Positively and if you find that this person has gone to take his or her drugs and you hear someone [nurse] like ‘your Antiretrovirals (ARVs) are there’. They feel de-motivated. They feel as if it is their fault. (Female, PE student)

#### Supports

Given these possibly stigmatising and discriminating scenarios, PE shared the experience that YLWHA would choose not to disclose their status or to disclose partially to a few trusted people within school. These would be mainly teachers, school administrators, PEs and trusted friends. This partial disclosure was esteemed by participants as a way for YLWHA to get psychosocial support such as comfort from friends, counselling from PEs, treatment support from school nurses as well as support from school administrators in form of school leave permits for them to attend Antiretroviral Therapy (ART) clinics or to stay home when they are sick. Support, however, would only be realised through self-disclosure rather than unintentional disclosure. Since anticipated and self-stigma would lead many YLWHA to choose not to disclose at all, they would miss possible psychosocial support at school since they would not be known and their needs would remain unknown too, as stated by some participants.

It is a student who came and talked to me about her status. She told me that in case you don’t see me, know that I am not feeling well. She said she was ok with the grand mum who was taking care of her. She wanted me to know her status in case she is not around, she either is not feeling well or has gone for her drugs, but she said the grand mum was caring. (Female, patron teacher)

### Health and treatment adherence

#### Stressors

The poor health and frequent illnesses were noted by participants as distresses to YLWHA, not solely giving rise to psychological distress as discussed before but also leading to poorer school attendance and academic performance in addition to dealing with suspicions among their peers regarding their health status.

According to participants, the school environment further avails a number of stressors to YLWHA which would impact their health and adherence to treatment. As already discussed in the two domains above, the lack of privacy and confidentiality at school, non-disclosure and fear of enacted stigma would cause YLWHA to hide drugs, take them in secrecy and sometimes to skip doses. They would therefore have no one to remind them and they would be unlikely to stick to their regimen. They would also refrain from seeking permission to leave school to attend to their clinic appointments.

Another problem is, if that person is in boarding school, when it comes to taking those tabs, the ARVs, it becomes a problem because the person may first hide them because he has no place to take them. So, the person ends up missing taking ARVs or skipping the dose of which it is bad. (Female, PE student)

You find conditions in school tell them that you have to keep your medicine with the nurse and of which sometimes they do not believe in the nurse, they cannot confide in her and they find it a challenge to keep their medicine which disturbs them a lot. (Male, PE student)

While providing support for coping with the psychological distress related to HIV/AIDS, the tight school schedule was also seen as a potential stressor that would cause YLWHA to forget their drugs or skip doses. It was stated that during examination periods, YLWHA would miss clinic appointments to fully write their examinations and those who would choose to attend the clinic would miss some examinations. In other instances, students would engage in ‘field’ activities that require them to spend two or more days away from school. With such activities, YLWHA would forget to carry or take their medicine due to change in the daily routine they are accustomed to. The periodic hassles of leaving or missing school to visit ART clinics to pick drugs and undergo some medical assessments would also challenge YLWHA. It would cause them to miss school activities in addition to feigning convincing reasons to merit school leave permits for those who may not have disclosed their status to the school authorities. Drug fatigue and knowing that drugs do not cure HIV were also noted as possible limitations to treatment adherence, although this was not unique to school-going youth.

One time we were with students for a trip and after reaching there, one girl told me that ‘madam I forgot my medicine at school’ and we were to spend there 2 days. (Female, patron teacher)

Another cited school stressor to health and treatment adherence was the school food. Participants were aware that taking ARVs requires adequate nutritious foods but noted that in many schools, students are fed on ‘posho’ (meal made from maize flour) and beans every day. Since these foods are detested by many school children including YLWHA, they would take drugs on empty stomachs leading to exacerbation of ARV-related side effects and possible rejection of drugs as suggested by participants. Those in day schools also may not have proper feeding due to the poor economic status of the households they come from. Some participants suggested that schools could provide special food to all YLWHA who would have disclosed their status to the school authority in order to promote treatment adherence, but many others were against this proposal as it would make YLWHA a special group in school, possibly exacerbating stigma.

#### Supports

Participants noted possibilities of support that schools could provide to YLWHA in the domain of health and treatment adherence. Despite the aforementioned challenges related to privacy and confidentiality, the presence of a school nurse was by participants seen as a measure to address adherence in school through reminders for YLWHA to take their drugs and safe custody of the drugs. PEs deemed it important that YLWHA should be encouraged to keep taking their drugs with the hope that a cure will be discovered given the enormous research and innovations in the medical field. According to them, teachers and PEs find themselves in the position of raising hope for YLWHA on the effects of treatment.

Through sensitisation, regular counselling and encouragement, school personnel and club members such as those of peer education clubs, debate clubs and science clubs could easily preach positive living that involves adherence to treatment. To further develop schools as supportive environments for YLWHA, participants suggested that in future, schools could choose a ‘guardian teacher’ for every YLWHA in school. This teacher would be responsible for all the support to the YLWHA including keeping their drugs and reminding them to take drugs in a private place provided by the guardian teacher.

They can give students with such a problem [HIV] a guardian teacher such that if they have problems, they can talk to that teacher. Such a teacher can even keep tabs [drugs] and remind you to take them. (Male, PE student)

PEs recommended school authorities to support YLWHA to adhere to treatment by providing school leave permits as well as arranging for them to leave school and seek treatment for other illnesses. Additionally, participants suggested that in future it would help if ARVs would be provided to YLWHA within school. This would eliminate the necessity to leave school and it would improve adherence through reminders and direct observation of drug consumption by the guardian teacher or school nurse as earlier suggested.

## Discussion

This study sought to understand the distressing and supportive features of the Ugandan school context that are deemed to influence the well-being of YLWHA in the aftermath of their seropositive diagnosis as well as the supports possibly available in the future to address their plight. We investigated daily stressors and supports for YLWHA in secondary schools in Kabarole district, western Uganda through the perspective of PE who are close and vital in facilitating support for YLWHA within a school setting.

Stigma appeared to run like a red thread through the peer educators’ analysis of what kind of an environment the school offers to YLWHA. It was a salient manifest and latent theme in all interviews and focus group discussions. Our findings allude to various forms of stigma that YLWHA would experience such as self-stigma (Arrey, Bilsen, Lacor, & Deschepper, 2015; Corrigan & Watson, 2002) perceived stigma (Earnshaw & Chaudoir, 2009), enacted stigma (Earnshaw & Chaudoir, 2009) and intersectional stigma (Logie, James, Tharao, & Loutfy, 2011). This confirms previous findings on the prominence of stigma in school contexts (Martinez, Lemos, & Hosek, and the Adolescent Medicine Trials Network, 2012; Michaud, Suris, Thomas, Gnehm, & Cheseaux, 2010; Mutumba et al., 2015; Orban et al., 2010) and corroborates literature on HIV/AIDS as a stigmatised health condition giving rise to stigmatised identities. Stigmatised identities can impact strongly on feelings about the self, social behaviour and health outcomes (Quinn & Earnshaw, 2013). The present study also showed that the stigmatised seropositive status and by extension identity was strongly perceived to intersect with youths’ psychological wellbeing, disclosure, and health and treatment adherence, which were in turn interrelated. As with concealable stigmatised identities like HIV/AIDS (Quinn & Earnshaw, 2013), the disclosure dilemma was in this study deemed a major challenge for YLWHA. They are confronted with an important but difficult decision of whether or not to disclose their serostatus to others. Winchester et al. (2013) referred to disclosure as ‘a double-edged sword’ due to its ability to garner psychosocial support while simultaneously creating stigma, shame and discrimination. The PEs portrayed disclosure of HIV by YLWHA to others in their environment as a ‘necessary evil’. They portrayed the school setting as a potentially rich social support environment because of the various potential support figures present, including school administrators, teachers, school nurses and fellow students. From the different social networks at school, YLWHA could be expected to have better psychosocial support in terms of comfort, counselling, medical adherence and nurturing of skills for positive living through sensitisation, as similarly reported by Murray, Crain, Meyer, McDonough, and Schweiss (2010). While the peer educators’ clubs and the PIASCY policy encourage openness about HIV/AIDS within schools so as to benefit from its supportive capacities, our findings are in line with other studies showing that prevailing stigma and lack of confidentiality are key-drivers to non-disclosure of YLWHA (Nabukeera-Barungi et al., 2015).

In this study and that of Abubakar et al. (2016), teachers were faulted for breaking confidentiality when they reveal the status of YLWHA who they would become aware of. YLWHA would therefore opt not to disclose to avoid stigma and discrimination (Chandra, Deepthivarma, & Manjula, 2003; Steward et al., 2008; Wolf et al., 2014) but forfeit the benefits of disclosure and bearing the burden of keeping a secret (Sandelowski, Lambe, & Barroso, 2004) amidst several pointers such as frequent illnesses, visible bodily signs and regular visits to the school nurse as also reported in this study. Studies on disclosure, adherence and stigma report that YLWHA have to keep lying about these suspicious pointers in their lives to maintain the secrecy (Abubakar et al., 2016). It has been noted that it is barely possible to maintain absolute secrecy in the school context (Abubakar et al., 2016; Mutwa et al., 2013). Secrecy thus forms a stressor that YLWHA have to bear with at a high psychological price since it requires vigilance during social interactions (Smart & Wegner, 2000).

As such, the supportive potential of school environments cannot be exploited fully due to stigma and non-disclosure typical of the school context. Mburu et al. (2013) found that stigma was an on-going challenge in Uganda, driven by social and economic factors. It was reported as a barrier to medical adherence and disclosure in Ugandan schools (Bikaako-Kajura et al., 2006). Several other studies have noted that stigma and the consequent non-disclosure form a major hindrance for secondary HIV prevention and care (Greeff et al., 2008; Wolf et al., 2014) and renders it difficult for schools to support HIV-positive students (Wolf et al., 2014). Similarly, in all interviews with PE, we identified that schools did not have programmes tailor-made for YLWHA since they tend to not know these students and their needs. We can therefore logically conclude that stigma currently limits the resources and supportive potential of school settings. PEs deemed partial disclosure (disclosure to a few trusted people) by YLWHA vital for them to get psychosocial support in form of comfort, reminders to take medicine, permit to leave school and attend to ART clinic appointments. Studies by King et al. (2008) and Wolf et al. (2014) identified similar benefits from partial disclosure, although their studies were not school-based and involved a broader population of people living with HIV/AIDS. Our findings also revealed some misconceptions about HIV and YLWHA in society that affect the way YLWHA are perceived in school. Future research should explore these misconceptions in the broader community so that they can be addressed for the benefit of schools as well.

PEs proposed that YLWHA could be assigned to special teachers they referred to as guardian teachers. Such teachers would preferably be positively living with HIV in order to act pillars for YLWHA within the school. A guardian programme in Tanzania to protect schoolgirls against sexual exploitation posted positive results (Mgalla, Schapink, & Ties Boerma, 1998). However, in the absence of documented evidence on the effectiveness of guardian teachers in HIV-care, more research is required to further examine this recommended strategy. We additionally foresee stigmatisation of guardian teachers due to their association with YLWHA. One prerequisite for this strategy to be beneficial for YLWHA, as derived from this study, is that the aforementioned disclosure and confidentiality concerns are addressed appropriately. In addition, well-designed and cautiously integrated stigma-reducing interventions are needed. These interventions should address the peculiar nature of HIV-stigma, which obviously reaches beyond the school setting. Although efforts to sensitise all people in Uganda about HIV/AIDS have been undertaken, minimal changes in attitude about HIV/AIDS and stigma have been recorded (Mburu et al., 2013). Our findings indicate that school environments offer the best platform for sensitisation of youth through mentorship programmes, clubs and visitations by external HIV/AIDS support organisations. At school young people can easily be reached with sensitisation messages as explained by Thomson, Currie, Todd, and Elton (1999).

Efforts to deal with HIV-stigma in Ugandan schools have been hitherto curricular based with messages discouraging discrimination and promoting care. We found, however, that teachers refrain from talking about HIV for fear of stigmatising YLWHA who may be in class, as also observed by Baggaley, Sulwe, Chilala, and Mashambe (1999). This shows that the principle of openness proposed in the Uganda National HIV/AIDS policy of 2011 is waning. Teachers should, therefore, be invigorated to boldly talk about HIV/AIDS in a non-stigmatising approach following teacher support interventions for improved communication about HIV/AIDS. It was also pointed out by some participants that HIV-prevention messages often were stigmatising to YLWHA. A similar concern was reported by Mudege and Undie (2009), in the formative evaluation of the PIASCY programme for Uganda. It is therefore imperative that such messages are scrutinised in a sensitivity check towards youth already living with HIV/AIDS.

Our study also pointed to the importance of extracurricular activities in supporting YLWHA in the school context, as PEs identified schools as providing better distractive avenues through extra-curricular activities such as clubs and games. These are enshrined in the school programmes and all students are encouraged to participate. These engagements at school were valuable for keeping YLWHA busy so that they do not think about their condition all the time thereby improving their psychological wellbeing. Studies by Mutumba et al. (2015) and Mutwa et al. (2013) also reported that YLWHA were able to cope by involving in distractive activities at school.

Besides the aforementioned psychological burden that keeping a secret may cause to YLWHA, it may also intersect with their health and treatment adherence according to PEs. Adherence to treatment among YLWHA was identified as another considerable daily stressor in the school setting, confirming earlier findings of Nabukeera-Barungi et al. (2015) and Mutwa et al. (2013). Participants reported possibilities of YLWHA hiding drugs, taking them in secrecy and encountering challenges to seek permission to leave school in order to attend to clinic appointments. This is linked to non-disclosure resulting from high perceived stigma and consequent lack of support. In order to promote positive living, the PIASCY policy advocates for adequate nutritious foods for people living with HIV such that they can maintain a healthy immune system. According to the PEs, this recommendation is currently not met in most secondary schools. The government of Uganda, through the Ministry of Health or Ministry of Education, could consider providing food supplements to YLWHA, which was found to significantly improve adherence to ART in a Zambian study (Cantrell et al., 2008). It was also suggested in our study and that of Mudege and Undie (2009) that adherence could be improved in future if ARVs are provided to students within school. Although the Uganda National HIV/AIDS policy calls for a multi-sectoral approach to address HIV/AIDS, treatment is a mandate of Ministry of Health. A policy shift towards ARV dispensing in schools would, therefore, be necessary for this to materialise.

### Study limitations and strength

We recognise that the qualitative design employed in this study and the small sample cannot allow generalizability of the findings but the selection of schools with varied students and geographical characteristics enabled us to obtain a broad perspective. Participants in the FGDs were also selected by patron teachers of the PEC. This could have introduced a selection bias as they could have selected the most active members. Moreover, we present perceptions of only PEC members who, although significant in the lives of YLWHA in school, are not the only stakeholders. A more substantiated picture could be obtained by involving other stakeholders such as other teachers, other students, HIV-positive students and school administrators in a large-scale study. However, we planned independent studies with other school stakeholders including YLWHA to gain depth into their perceptions. We also admit that observational approaches would be necessary for some aspects of this study to corroborate the views of the participants.

### Conclusion

The school provides a potentially supportive environment for the wellbeing of YLWHA, which, however, has not been exploited fully due to concerns about confidentiality and stigma hindering disclosure and treatment adherence. As a result, participants reported more stressors than support as presented in the findings. To foster disclosure in a non-discriminatory and stigma-free environment and to support psychological well-being and treatment adherence, this study recommends measures that increase resilience within the school community to deal with special needs of YWLHA. Such measures should involve all stakeholders and should have a major focus on tackling stigma. This calls for concerted efforts from all school stakeholders and government line ministries. Societies that serve and are served by the schools are instrumental stakeholders that shape ideas, beliefs and practices in schools for schools are not islands.
